# Prognostic Value of Platelet to Lymphocyte Ratio in Sepsis: A Systematic Review and Meta-analysis

**DOI:** 10.1155/2022/9056363

**Published:** 2022-06-06

**Authors:** Gancheng Wang, Azam Mivefroshan, Shirin Yaghoobpoor, Shokoufeh Khanzadeh, Goli Siri, Farzad Rahmani, Samira Aleseidi

**Affiliations:** ^1^Department of Biomedical Engineering, College of Engineering, Peking University, Beijing, China; ^2^Department of Adult Nephrology, Urmia University of Medical Sciences, Urmia, Iran; ^3^Student Research Committee, Faculty of Medicine, Shahid Beheshti University of Medical Sciences, Tehran, Iran; ^4^Student Research Committee, Tabriz University of Medical Sciences, Tabriz, Iran; ^5^Department of Internal Medicine, Amir Alam Hospital, Tehran University of Medical Sciences, Tehran, Iran; ^6^Emergency Medicine Research Team, Tabriz University of Medical Sciences, Tabriz, Iran; ^7^Rheumatology Research Center, Tehran University of Medical Sciences, Tehran, Iran

## Abstract

The goal of this study was to conduct a systematic review of the literature on the relationship between peripheral blood platelet to lymphocyte ratio (PLR) and mortality in sepsis and to integrate the findings in a meta-analysis. An electronic search of three main databases was performed: PubMed, Embase, and Scopus on 19 December 2021. Finally, 16 studies comprising 2403 septic patients, including 1249 survivors and 1154 nonsurvivors, were included in this meta-analysis. We found that PLR levels were significantly higher in nonsurvivors than in survivors (random effect model: SMD = 0.72, 95% CI; 0.35–1.10, *p* < 0.001). However, significant heterogeneity was observed across the studies (*I*^2^ = 94.1%, *p* < 0.01). So, we used random effect model in our meta-analysis. In the subgroup analysis, according to mortality time, patients deceased during one month after sepsis had elevated levels of PLR compared to survivors (SMD = 1.03, 95% CI = 0.15-1.92, *p* = 0.22). However, in-hospital mortality was not associated with PLR level (SMD = 0.41, 95% CI = −0.18-0.99, *p* = 0.175). Our findings support PLR to be a promising biomarker that can be readily integrated into clinical settings to aid in the prediction and prevention of sepsis mortality.

## 1. Introduction

Sepsis is a complicated condition caused by a malfunction of the host's immune response to infection, which results in an uncontrollable inflammatory response and immunosuppression [1]. It develops as a result of infections acquired both in the community and in the healthcare system, particularly in intensive care units (ICUs), where it is the leading cause of death, responsible for more than half of all ICU deaths. Consequently, sepsis is seen as a global health problem with significant economic effects [1, 2]. As a result, identifying prognostic and diagnostic biomarkers is critical in order to avoid adverse outcomes and reduce mortality by initiating treatment before irreversible damage occurs. A delay of one hour in sepsis treatment is thought to be associated with a 7–10 percent increase in sepsis-related death [2]. As a result, many efforts have been made to find a viable biomarker for screening sepsis patients who are at a high risk of death. Among all the sepsis biomarkers studied, complete blood count (CBC) metrics, including the neutrophil to lymphocyte ratio (NLR) and platelet to lymphocyte ratio (PLR), could be valuable tools [1]. Undoubtedly, CBC has many advantages: (i) it is inexpensive, (ii) it has a quick turn-around time (TAT), (iii) it is accessible in all health centers, (iv) it is simple to perform, (v) clinicians regularly request CBC as part of patient management, and (vi) it is the most commonly ordered laboratory test in all medical settings, from the ICU to the emergency department (ED) [1]. A recent meta-analysis showed that nonsurvivors had significantly higher NLR levels than survivors (random effect model: SMD = 1.18, 95% CI = 0.42–1.94). They also looked at the predictive value of NLR in patients with sepsis; the results showed that increased NLR was linked to a bad prognosis in sepsis patients (fixed-effects model: HR = 1.75, 95% CI = 1.56–1.97) [3]. They came to the conclusion that NLR could be a useful predictive biomarker for sepsis patients, with greater NLR values indicating a worse prognosis. On the other hand, PLR, as an integrated reflection of thrombotic/inflammatory pathways, has been demonstrated to have predictive significance in a variety of human diseases, including cardiovascular disease [4], stroke [5], and cancer [6]. According to a growing number of studies, platelets are implicated in the pathophysiological pathways of sepsis and play a key role in organ dysfunction. Platelet activation is induced by inflammatory-coagulation reactions in sepsis and damaged endothelial cells, and these activated platelets can worsen coagulation disorders and systemic inflammatory reactions [7]. Low lymphocyte numbers may also be linked to a lower survival time in sepsis [8]. Indeed, lymphopenia is a common hallmark of sepsis-induced immunosuppression, as it prevents microbial clearance and predisposes to serious infections, which are the leading cause of sepsis-related death [8]. As a result, it has been suggested in previous research that an elevated PLR is indicative of an elevated host thrombotic/inflammatory response linked to sepsis mortality [9–24]. The goal of this study was to conduct a systematic review of the literature on the relationship between peripheral blood PLR and mortality in sepsis and to integrate the findings in a meta-analysis. Our hypothesis was that a high PLR is associated with a high mortality rate and that it might thus be used as a simple and cheap prognostic marker in general practice and for patient stratification in clinical trials.

## 2. Material and Method

Our systematic review and meta-analysis were performed in accordance with guidelines for the Preferred Reporting Items for Systematic Reviews and Meta-Analyses.

### 2.1. Data Sources and Searches

An electronic search of three main databases was performed: PubMed, Web of Science, and Scopus on 19 December 2021. The search terms included ((platelet AND lymphocyte AND ratio) OR (platelet-to-lymphocyte) OR PLR) AND (sepsis OR septic OR bacteremia) AND (mortality OR prognosis OR outcome OR surviv∗). Reference lists of retrieved articles were investigated to find more relevant articles.

### 2.2. Study Selection

Inclusion criteria were as follows: (i) studies in sepsis assessing the prognostic role of the peripheral blood PLR, (ii) availability of a mean and standard deviation (SD) of PLR or median (interquartile range (IQR)) or median (range) from which mean and standard could be calculated, and (iii) peer-reviewed journal articles of which full texts were published. Exclusion criteria were as follows: (i) studies involving animals, cell lines, or human xenograft experiments; (ii) case series, case reports, or review articles; (iii) duplicate publications; and (iv) studies in which PLR data were presented as odds ratio (OR), hazard ratio (HR), or risk ratio (RR) instead of mean and SD.

All of the articles found by the search strategy were examined independently by two reviewers (S.K. and S.Y.). The consensus was used to resolve disagreements. After excluding duplicate articles and obviously irrelevant articles, the full text of all possibly relevant papers was retrieved and evaluated for eligibility. Any missing or confusing data was clarified by contacting the corresponding author.

### 2.3. Endpoints of Interest

Survival prediction based on PLR value was the outcome of interest. So, we compared the survivor and nonsurvivor septic patients in PLR levels.

### 2.4. Data Extraction

Predesigned abstraction forms were used for data collection by two authors (S.K. and S.Y.) independently. The consensus was used to settle disagreements. The following data were extracted: name of the first author, year of publication, study location, age group (children or adults), mortality time assessed in the article, article language, collection of data (prospective or retrospective), race (white or East Asian), number of survivors, and nonsurvivors, as well as their PLR levels. We considered the patients from Turkey, Serbia, Poland, India, Iran, and Saudi Arabia as white people and patients from Korea, China, and Indonesia as East Asian people.

### 2.5. Quality Assessment

The Newcastle-Ottawa Quality Assessment Scale (NOS) [25] including three sections of selection, comparability, and outcome was used to evaluate and score the methodological quality of included studies. High-quality studies had a score of 6 or higher.

### 2.6. Data Synthesis and Statistical Analyses

The standard mean difference (SMD) with 95% CI was used instead of the weighted mean difference (WMD) to account for differences in PLR measuring procedures between investigations. Subgroup analyses were also conducted on the basis of mortality time (one-month mortality, in-hospital mortality), study design (retrospective, prospective), age group (children, adult), and race (white, East Asian). Due to significant heterogeneity between studies, a random effect model was adopted in our meta-analysis. Statistical heterogeneity was assessed using *I*^2^ statistics and Cochran's *Q* test. We used the method introduced by Wan et al. to estimate mean and SD from median (IQR and/or range) [26]. Publication bias was determined using Egger's test *p* value and visual inspection of funnel plots. Statistical significance was conceived as *p* < 0.05, and all statistical tests were two-sided.

## 3. Results

### 3.1. Identification of Relevant Studies

**Figure 1:**
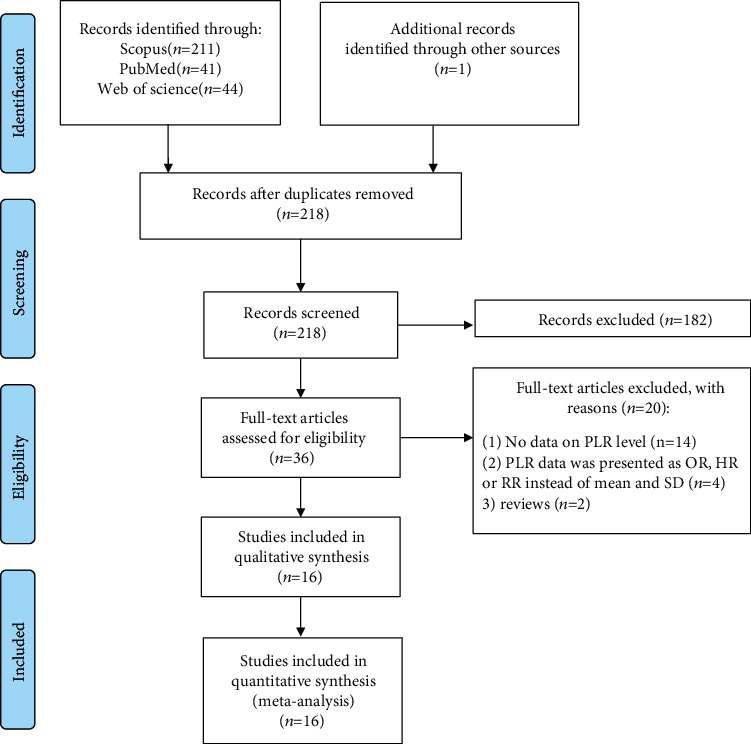
Flow chart of search and study selection.

The initial literature search retrieved 257 potentially eligible studies based on the predefined selection criteria. After eliminating the duplicates, we selected 36 studies through screening the titles and abstracts. After a detailed evaluation of the full texts, 20 studies were excluded, including 14 with insufficient data, two that were reviews, and four studies in which survival was compared between high versus low PLR group and reported OR or HR or RR instead of mean and SD. Thus, 16 studies [9–24] comprising 2403 septic patients, including 1249 survivors and 1154 nonsurvivors, were included in this meta-analysis.  Figure 1 shows a flow chart summarizing the selection process.

### 3.2. Study Characteristics and Quality Assessment

Among the 16 included studies, nine studies had prospective designs. Studies were conducted in China (*n* = 4) [12, 22–24], Indonesia (*n* = 3) [16, 19, 20], Turkey (*n* = 3) [10, 11, 18], Iran (*n* = 1) [14], Serbia (*n* = 1) [13], Saudi Arabia (*n* = 1) [9], Poland (*n* = 1) [17], Korea (*n* = 1) [15], and India (*n* = 1) [21]. Seven studies reported one-month mortality [9, 10, 12, 15, 19, 21, 24], three studies reported in-hospital mortality [13, 14, 22], and one study investigated ICU mortality [17]. Five studies did not report any data in this regard [11, 16, 18, 20, 23]. The population in the two studies was septic children [16, 20] and in 14 studies were adult septic patients [9–15, 17–19, 21–24]. Seven studies were retrospective [9–11, 17, 18, 20, 22], and nine were prospective [12–16, 19, 21, 23, 24]. The quality of the studies was high, with scores ranging from 7 to 9. The general characteristics of the patients in each study are listed in Table 1.

### 3.3. Comparison of PLR between Survivors and Nonsurvivors

**Figure 2:**
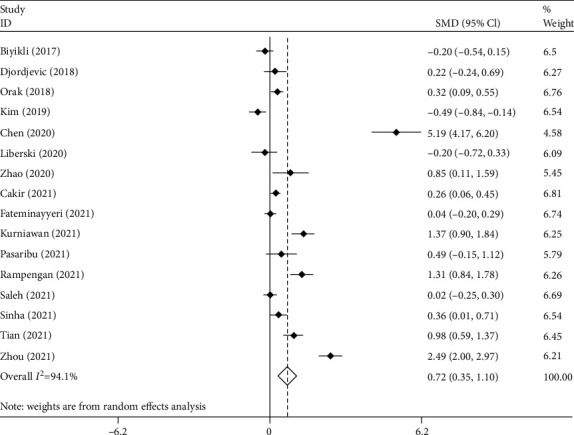
Meta-analysis of differences in PLR level between survivor and nonsurvivor septic patients.

After polling the data of 16 studies [9–24] with 2403 septic patients, including 1249 survivors, we found that PLR levels were significantly higher in nonsurvivors than in survivors (random effect model: SMD = 0.72, 95% CI; 0.35–1.10, *p* < 0.001). However, significant heterogeneity was observed across the studies (*I*^2^ = 94.1%, *p* < 0.01; Figure 2). So, we used random effect model in our meta-analysis.

**Figure 3:**
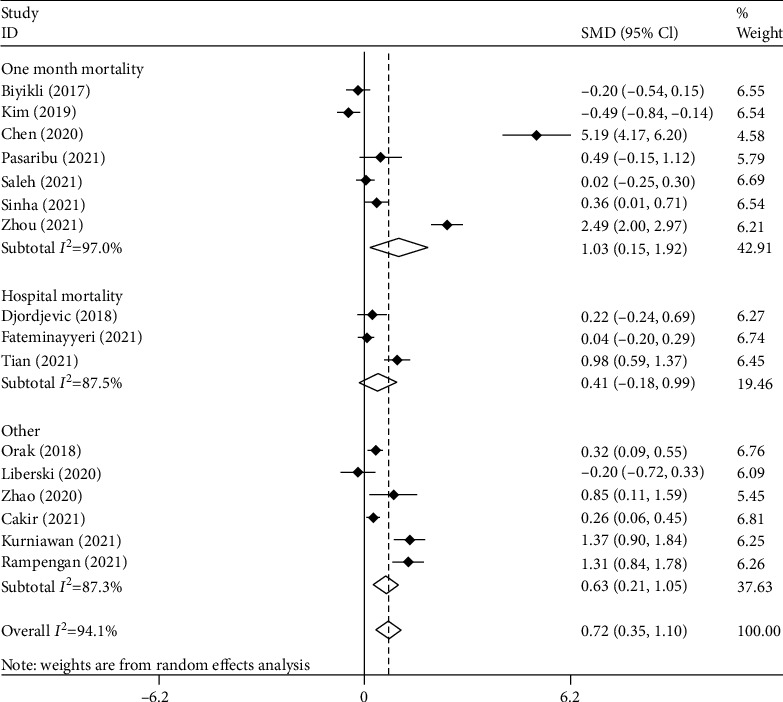
Subgroup analysis of differences in PLR level between survivor and nonsurvivor septic patients according to mortality time.

In the subgroup analysis, according to mortality time reported in articles, there were seven studies on one-month mortality [9, 10, 12, 15, 19, 21, 24], including 850 septic patients, of which 469 survived, and three studies on in-hospital mortality [13, 14, 22], including 1553 septic patients of which 780 survived. As seen in Figure 3, patients deceased during one month after sepsis had elevated levels of PLR compared to survivors (SMD = 1.08, 95% CI = 0.15-1.92, *p* = 0.22). However, in-hospital mortality was not associated with PLR level (SMD = 0.41, 95% CI = −0.18-0.99, *p* = 0.175).

**Figure 4:**
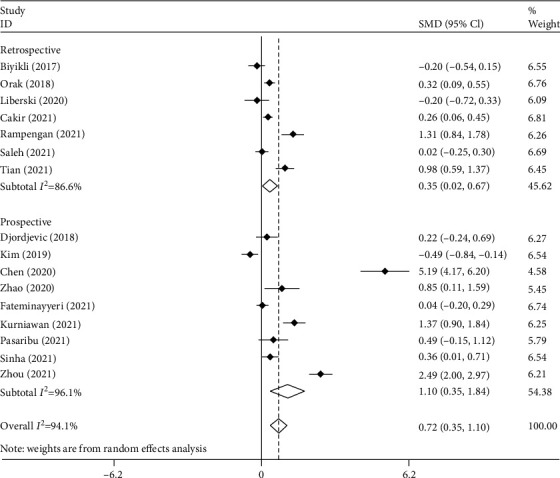
Subgroup analysis of differences in PLR level between survivor and nonsurvivor septic patients according to study design.

Figure 4 shows the subgroup analysis according to the study design. We found seven retrospective studies [9–11, 17, 18, 20, 22], including 1419 septic patients with 692 survivors and nine prospective studies [12–16, 19, 21, 23, 24] including 984 septic patients with 557 survivors. Nonsurvivors had elevated levels of PLR compared to survivors in either retrospective (SMD = 0.35, 95% CI = 0.02-0.67, *p* = 0.035) or prospective studies (SMD = 1.10, 95% CI = 0.35-1.84, *p* = 0.004).

**Figure 5:**
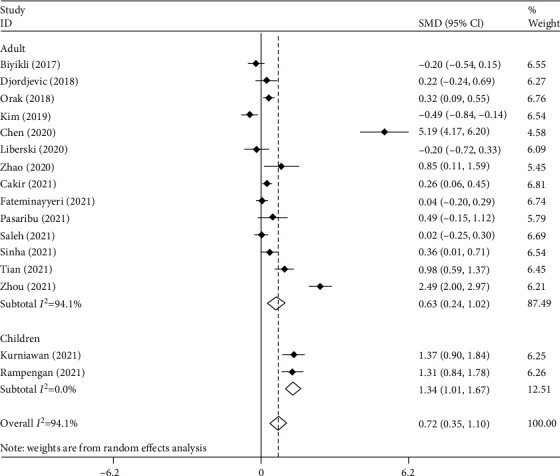
Subgroup analysis of differences in PLR level between survivor and nonsurvivor septic patients according to age group.

In another subgroup analysis according to age group, there were two studies on septic children [16, 20] comprising 174 patients, of which 87 survived, and 14 studies on adult septic patients [9–15, 17–19, 21–24] with 2229 patients including 1162 survivors. As shown in Figure 5, PLR levels were higher among nonsurvivors compared to survivors in either child (SMD = 1.34, 95% CI = 1.01-1.67, *p* = 0.001) or adult groups (SMD = 0.63, 95% CI = 0.24-1.02, *p* < 0.001).

**Figure 6:**
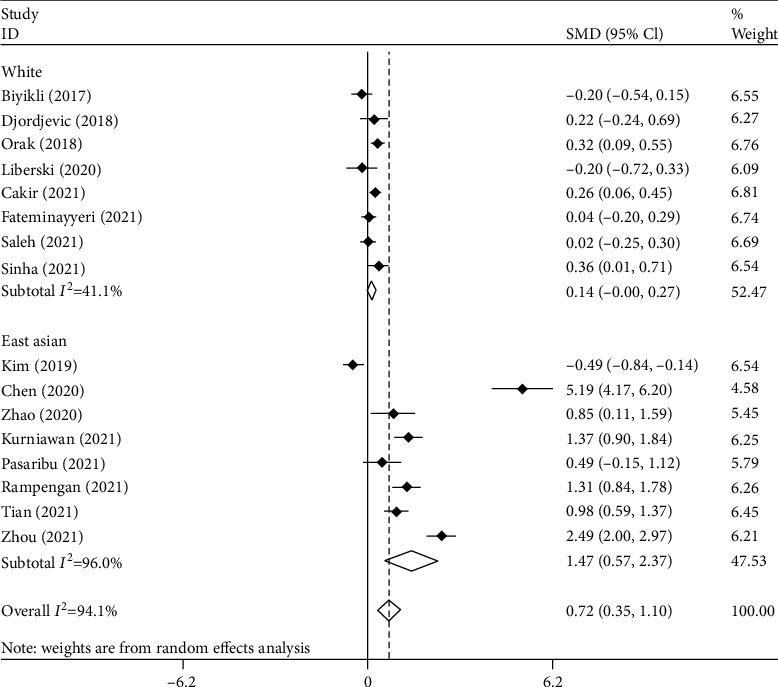
Subgroup analysis of differences in PLR level between survivor and nonsurvivor septic patients according to race.

Figure 6 presents the final subgroup analysis according to race, including eight studies on white people [9–11, 13, 14, 17, 18, 21] with 1610 patients including 738 survivors and eight studies on East Asian people [12, 15, 16, 19, 20, 22–24] with 793 patients including 511 survivors. PLR levels were higher among nonsurvivors compared to survivors in the East Asian group (SMD = 0.72, 95% CI = 0.35-1.10, *p* = 0.001) but not in the white group (SMD = 0.14, 95% CI = −0.00-0.27, *p* = 0.052).

**Figure 7:**
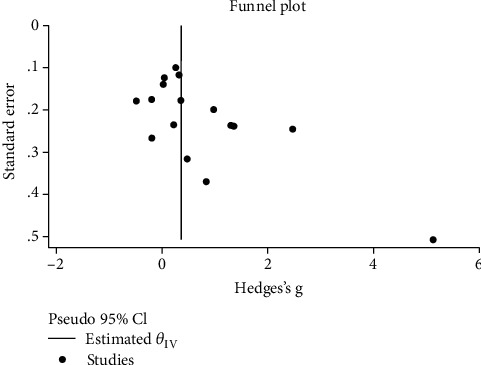
Funnel plot assessing publication bias.

As seen in Figure 7, there was some indication of publication bias among studies on the role of PLR in sepsis (Egger's test *p* = 0.001). However, exclusion of one outlying study [12] from the analysis attenuated Egger's test to nonsignificance (*p* = 0.12).

## 4. Discussion

Sepsis and septic shock are two of the most common causes of death worldwide, and they come with high treatment expenses [27]. Mortality prediction is a significant issue in sepsis management. In sepsis patients, laboratory parameters or biomarkers are utilized to diagnose and predict the clinical outcomes [28]. Multiple biomarkers have been evaluated in the hopes of aiding prognosis and diagnosis. Still, none of them have proven accurate enough to be utilized in routine daily clinical cases. During recent years, lymphocyte and platelet counts have been discovered to play essential roles within the inflammation reaction [29]. As a result, PLR has been studied as a possible biomarker of inflammation in a number of disorders, particularly sepsis [30]. For example, the PLR has been correlated to the diagnosis, monitoring, and prognosis of tumors in the digestive, reproductive, and respiratory systems [31].

In this study, we applied a meta-analysis in order to combine 18 studies to investigate whether PLR can be a potential prognostic biomarker in sepsis. The main result of the current systematic review and meta-analysis study was that PLR among sepsis nonsurvivors was significantly higher than the sepsis survivors. In order to attain a comprehensive explanation for PLR as a prognostic biomarker in patients with sepsis, it is required to figure out the roles of lymphocytes and platelets in sepsis.

During sepsis and severe injuries, including burns, trauma, and major surgeries, apoptosis-induced lymphopenia is common. This process starts immediately after the underlying damage occurs. The severity and length of lymphopenia are associated with poorer clinical outcomes, such as preceding infections and higher mortality rates. Apoptosis is among the most likely reasons for injury-related lymphopenia, and it plays a role in injury-induced immunoparalysis both directly and indirectly. In response to diverse insults, the immune system causes a rise in neutrophil count and a reduction in lymphocyte count. Lymphocyte count drops because active lymphocytes migrate to inflamed areas, and lymphocyte apoptosis rises [32]. Clinical studies have shown that lymphocyte counts in the blood fall with the onset of sepsis and stay low for up to 28 days [33–39].

Many septic patients develop prolonged and severe immunosuppression before dying to the disease, after an initial preponderance of a proinflammatory cytokine-driven reaction [36, 40–42]. The findings show that a persistent low circulating lymphocyte on the fourth day after a sepsis onset predicts short-term and long-term survival independently and could be used as a biomarker for sepsis-induced immunosuppression. The immune response to sepsis is exceedingly varied, and it can alter significantly as the condition worsens. Patients who die at early stages do so due to severe hyperinflammation, which manifests itself as multiple organ failure and cardiovascular collapse [43, 44]. Patients who stay alive at this stage represent a compensating anti-inflammatory response accompanied by more inhibitory receptors on T cells and antigen-presenting cells, reduced proinflammatory cytokine secretion, increase in myeloid-derived suppressor cells, and apoptosis-related death of lymphocyte and dendritic cells [41, 45–48]. Drewry et al. found that while both sepsis survivors' and nonsurvivors' absolute lymphocyte counts decline to low numbers at the initiation of sepsis, nonsurvivors' absolute lymphocyte counts continue to stay persistently low while there is a recovery in the survivors' lymphocyte counts [8].

Sepsis is coupled with a malfunction of the hemostatic system, and platelets play a key role in both hemostasis and the immune response to diverse insults [13]. According to many studies, platelets are implicated in the pathophysiology of sepsis and play a key role in organ damage [49, 50]. After the invasion of pathogens into the body, the coagulation system is activated at the site of the infection, and thrombus is produced in local capillaries in order to serve as protective mechanisms aiming to limit infection to the lesions [51]. In sepsis, these local reactions propagate throughout the body, and a lack of control of the “inflammation-coagulation” interaction leads to disseminated intravascular coagulation (DIC) and multiorgan failure syndrome (MODS). In sepsis, platelet activation is triggered by inflammation-coagulation interactions and endothelial cell damage. Activated platelets can aggravate systemic inflammatory reactions and coagulation abnormalities through interactions with endothelial and inflammatory cells and other mechanisms [52–54]. Platelets also have Toll-like receptors (TLRs), which allow them to distinguish different molecular patterns of bacteria, and platelets can become activated as a result of this [13]. Platelets secrete thromboxanes and other mediators, leading to more significant inflammation among patients with a high platelet count [55].

In the bone marrow, platelets are generated by mature megakaryocytes. Recent research has found that cytokines such as thrombopoietin (TPO), IL3, IL6, IL9, IL11, and stem cell factor (SCF) can increase megakaryocyte production [56]. These factors are found to be increased in septic patients. This may link the increased PLR to sepsis severity.

It is indicated that levels of IL-6 are increased in septic patients [29, 57], and it could be used as a predictor of survival [57, 58]. IL-6 also promotes the conversion of megakaryocytes to platelets and is implicated in neutrophil recruitment [59]. Levels of IL-3, another inducer in megakaryocytes production, are also higher in patients with sepsis and correlate with the severity of disease [60, 61]. Also, Froeschle et al. reported a higher level of IL-9 in neonatal septic patients. This cytokine plays a crucial role in neonatal sepsis [62].

TPO directly affects the homeostatic potential of mature platelets as well as its function in thrombopoiesis. TPO, for instance, promotes platelet activation and platelet-leukocyte adhesion in response to several agonists, despite its inability to stimulate platelet aggregation [56, 63, 64]. Several studies have shown TPO levels to be increased following endotoxin infusion in healthy individuals [65], and in septic neonates, children [66–68], and adults [69, 70]. According to a study by Segre et al., TPO levels were positively correlated with sepsis severity [71]. Lupia et al. discovered a link between TPO levels and platelet activation in patients with burn injuries, mainly when septic complications arise [72], a characteristic that can lead to microthrombotic events and worsen organ damage [73]. Furthermore, another study by Lupia et al. discovered that TPO collaborates with TNF and IL-1 to mediate the negative cardiac inotropic effect induced in vitro by serum samples of patients with septic shock [73]. Therefore, increased TPO levels during sepsis may augment platelet activation and play a role in the pathophysiology of multiorgan failure in such a pathological state.

The imbalance between the two cells is reflected in the PLR change. In this case, an increase in the PLR suggests an imbalance in the proinflammatory and anti-inflammatory reactions. This immune response imbalance causes numerous organ failures, metabolic problems, immunodeficiency, and a mismatch between oxygen supply and demand, all of which lead to mortality [12]. In sepsis, the immune response involves both pro- and anti-inflammatory activities simultaneously, and the immune response is often separated between a main cytokine-mediated hyperinflammatory stage and a secondary immuno-suppressive stage [74, 75]. Numerous proinflammatory cytokines are produced during the hyperinflammatory phase [75], which cause neutrophilia, lymphopenia, and platelet formation in the bone marrow, resulting in a rise in PLR. Platelets secrete inflammatory cytokines and interact directly with bacteria and cells in the body, particularly neutrophils, T lymphocytes, NK cells, and macrophages. These immune cells contribute to the worsening of inflammation. Meanwhile, low lymphocyte numbers imply immunological suppression. This implies that a high platelet count shows significant inflammation, whereas a low lymphocyte count suggests a poor immunological response to infection. As a result, increased PLR levels are related to severe systemic inflammation and can worsen some conditions, such as sepsis [29].

## 5. Limitations

Our study has a few limitations that are important to address. It is important to note that PLR values vary according to race, and such variations may explain the lack of significance in geographic subgroup analysis for PLR. It is possible that certain populations may not experience characteristic alterations in hematopoiesis following critical illness, and thus, PLR may not have utility in associated geographic regions. Further, inherent to a meta-analysis is a risk for study heterogeneity. High heterogeneity could be due to the fact that among selected studies, more than one method was used to diagnose sepsis, and among those used, there is also a risk for user variability due to their subjective nature. In addition, there was a significant publication bias which we tried to explain its source.

## 6. Conclusion

In conclusion, inflammation is strongly connected with the PLR levels, making it a helpful biomarker for predicting the severity of an inflammatory process such as sepsis. A high PLR value implies a more severe inflammatory response. Clinical worsening, a worse prognosis, and mortality could result from more severe inflammation. Our study indicated that PLR levels among sepsis nonsurvivors are significantly higher than the survivors. Therefore, PLR is a low-cost and straightforward potential clinical predictor that can be employed even in resource-constrained settings. Further research is needed to investigate the sensitivity and specificity of PLR as a definite prognostic biomarker in sepsis.

## Figures and Tables

**Table 1:** General characteristics of included studies.

Author	Year	Mortality time	Design	Age group	Country	Race	Survivor		Nonsurvivor		NOS
							*N*	NLR	*N*	NLR	
Biyikli	2017	30 days	R	Adults	Turkey	White	72	207.60 ± 189.63	59	168.31 ± 209.83	8
Djordjevic	2018	In-hospital	P	Adults	Serbia	White	57	226.95 ± 145.00	26	260.57 ± 165.64	8
Orak	2018	NA	R	Adults	Turkey	White	111	240.97 ± 171.54	219	364.05 ± 452.56	7
Kim	2019	One month	P	Adults	Korea	East Asian	114	331.77 ± 268.07	44	202.70 ± 253.63	9
Chen	2020	28 days	P	Adults	China	East Asian	26	115.00 ± 14.00	41	208.00 ± 20.00	9
Liberski	2020	ICU	R	Adults	Poland	White	21	354.96 ± 261.16	40	298.76 ± 300.03	8
Zhao	2020	28 days	P	Adults	China	East Asian	30	152.84 ± 87.13	10	238.64 ± 135.25	9
Cakir	2021	NA	R	Adults	Turkey	White	182	178.74 ± 202.99	229	251.84 ± 331.59	7
Fateminayyeri	2021	In-hospital	P	Adults	Iran	White	130	349.00 ± 617.00	130	376.00 ± 617.00	8
Kurniawan	2021	NA	P	Children	Indonesia	East Asian	50	77.54 ± 50.08	37	157.13 ± 67.38	7
Pasaribu	2021	28 days	P	Adults	Indonesia	East Asian	18	148.54 ± 50.08	22	296.09 ± 299.79	9
Rampengan	2021	NA	R	Children	Indonesia	East Asian	37	77.54 ± 50.08	50	157.13 ± 67.38	7
Saleh	2021	28 days	R	Adults	Saudi Arabia	White	107	225.31 ± 154.33	98	230.81 ± 301.35	9
Sinha	2021	28 days	P	Adults	India	White	58	22.54 ± 20.42	71	36.60 ± 49.58	9
Tian	2021	In-hospital	R	Adults	China	East Asian	162	185.76 ± 105.31	32	354.63 ± 355.52	8
Zhou	2021	NA	P	Adults	China	East Asian	74	83.72 ± 10.33	46	114.65 ± 15.26	7

NA: not applicable; R: retrospective; P: prospective; NLR: neutrophil to lymphocyte ratio; N: number; ICU: intensive care unit.

## Data Availability

All data generated or analysed during this study are included in this published article.
